# Unexpectedly strong hydrophilic character of free-standing thin films from carbon nanotubes

**DOI:** 10.1038/s41598-017-12443-y

**Published:** 2017-09-25

**Authors:** Dawid Janas, Grzegorz Stando

**Affiliations:** Department of Chemistry, Silesian University of Technology, B. Krzywoustego 4, 44-100 Gliwice, Poland

## Abstract

We report on the development of a method of formation of hydrophilic carbon nanotube (CNT) films. The technique is simple, straightforward and does not require specialized equipment or use of harsh chemical compounds. Elimination of the need for oxidizing agents has paramount implications because it preserves the inherent CNT properties. A reference study, in which the traditional method of oxidation of CNTs was used to introduce functional groups, gave smaller reduction of water contact angle and made a negative influence on the surface chemistry. From the practical point of view, this method is an important step towards implementation of CNTs in the real life by making them more compatible with interface materials. Interestingly, the method gives high level of control over the surface character of CNT films and hydrophilic character can be precisely patterned where required.

## Introduction

Carbon nanotubes (CNTs) have shown high performance in a wide range of applications ranging from thermal^1–4^, electrical^5–7^, mechanical^8,9^ to many other^10–12^. Due to the excellent properties of individual CNTs, they often outperform traditional materials such as copper or aluminium commonly used at an industrial scale. There are however some issues, which must be taken care of, before we can start implementing these carbon nanostructures in the real life. One of the problems is that it is difficult to translate the properties from the nanoscale onto their macroscopic assemblies, which consist of millions of individual CNTs, such as fibers or films^7,13^. Poor alignment and contact resistance between individual CNTs in such aggregate results in phonon or charge carrier scattering, which significantly deteriorates the performance in terms of electrical or thermal conductivity^14–16^. Moreover, topological defects have a strong influence on the mechanical properties and even a small number of them can greatly decrease their strength^17^. For CNTs to show competitive advantage over commonly used materials these issues must be addressed.

Another important aspect is poor compatibility of CNTs with many materials due to high total surface free energy (72.9 mJ/m^2^ ^18^). The consequence is that finding appropriate interface material as protective outer layer (*e.g*. for electrical insulation of CNTs) is challenging. Metal or polymer matrices do not interact well with as-made CNTs, therefore the most common approach involves chemical^19–21^ or physical functionalization^22^. Such processing often involves reactive chemical species (*e.g*. H_2_SO_4_:HNO_3_ mixtures) that introduce hydrophilic functional groups, which disrupt the delicate network of sp^2^ carbon atoms. Prolonged or more vigorous treatment actually may cut the individual CNTs into pieces, which indicates how difficult to control these methodologies are. On the other hand, physical functionalization such as polymer wrapping, which appears more benign, separates CNTs from one another.

Here we present a convenient method of manufacturing free-standing CNT films with high affinity towards hydrophilic media. The examination of contact angles of various liquids revealed that the straightforward strategy was successful and the observed effect was to a large extent permanent in time. Most importantly, encouraging results were obtained without strong functionalization of the material what preserved their inherent nature. A reference study in which we tested CNT films made from CNTs oxidized by harsh chemical compounds gave smaller reduction of water contact angle, what once again demonstrates the viability of the proposed methodology. To prove the concept we demonstrated how the precisely engineered hydrophilic CNT films have interact with polymer matrices.

## Experimental

Three types of as-made CNTs were used for the study: technical grade Nanocyl NC7000 (NC) and produced in house: CNT carpet (CPT) and *N*-doped CNT carpet (*N*-CPT). For the synthesis, the conditions based on published research methodologies were employed^23,24^. In brief, 5.5 wt% solution of ferrocene in toluene (catalyst and carbon source) was injected into a horizontal furnace kept at 760 °C at the flow rate of 4 mL/h over the course of 5 hours. The reaction was carried out under the protective flow or argon (1.8 L_normal_/min). Upon completion, CPT material was scraped off quartz reaction tube. For the *N*-CPT synthesis, 1:9 V/V equivalent of pyrimidine with respect to toluene was added to the reaction mixture. In addition, single-wall CNTs Tuball purchased from OCSiAl were used as reference.

NC and CPT materials were also subjected to a range of oxidation treatments, which are thoroughly described in Supplementary Information file (piranha solution, H_2_SO_4_/HNO_3_, H_2_O_2_, KMnO_4_ exposure). The most promising samples were processed into CNT films to evaluate their wettability by contact angle measurements.

**Figure 1:**
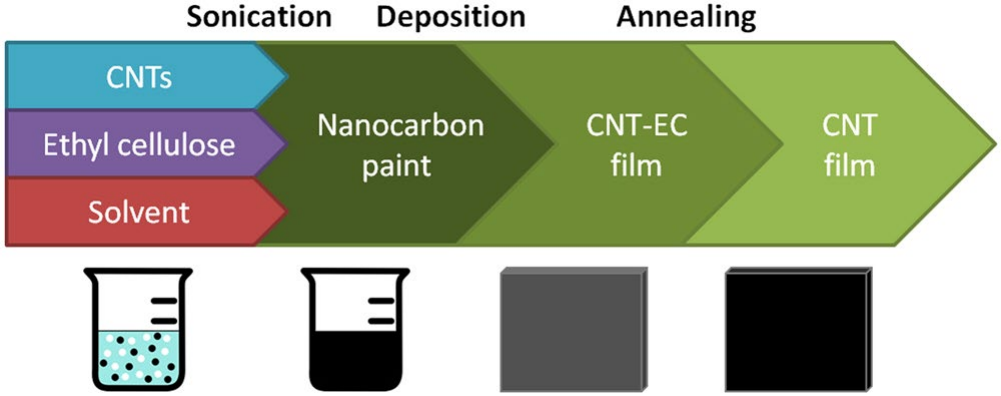
Free-standing CNT film manufacture.

The CNT films were produced by a previously reported method (Fig. 1)^25^. In brief, CNTs and ethyl cellulose (EC) were dispersed in acetone/toluene mixture by means of ultrasonication. As-formed CNT paint was deposited on a substrate (Kapton sheet), from which it was detached due to low affinity of the nanofilm to the substrate. As a final step, where indicated, binder was removed by igniting the film with a lighter (referred to as flash annealing) to give a CNT film free of polymer. CNT films for further investigations were produced from the following materials: neat CPT, *N*-CPT and NC, as well NC and CPT both treated by piranha solution at 130 °C for 3 h.

As a reference for contact angle measurements, EC film was produced. 1 g of EC was dissolved in 20 mL of toluene and then dripped onto 25 mm × 75 mm glass slides. Once it was dried in the ambient, next layer of EC was applied. In total 5 layers were applied to produce an EC film.

Initial contact angle measurements were carried out using model liquids (0.05 mL droplets) of to test their affinity towards CNT films: water and hydrophilic, but much more viscous, glycerol were employed.

For flash annealed NC films, evolution of water contact angle in time was registered to investigate how permanent is the character of the surface properties. We prepared two sets of samples. One batch was used repeatedly, in the other case fresh CNT films were employed every time. Both batches were stored in the ambient. Water contact angle was registered 5 min after droplet application, which took place in day 0, 1, 4, 8, 11, 14 and 30. The average humidity was 70% during May 2017, when these experiments were carried out. For all contact angle measurements, results from 10 samples were averaged in order to get a reliable value.

To prove compatibility of processed carbon nanomaterials with polymer matrices, contact angle of Bisphenol A diglycidyl ether (D.E.R. 332) was also recorded in time for particular materials. 1 droplet of epoxy resin was deposited on each fresh CNT film (as-made or annealed) to observe its contact angle evolution. 5 CNT films were used for each case.

The hybrid hydrophilic-hydrophobic film was created by annealing a half of 20 mm × 40 mm CNT&EC film. That half was covered with a microscope glass slide, which protected EC from combustion, and so preserved its hydrophobic character.

Scanning Electron Microscopy (SEM, FEI Nova NanoSEM) and Transmission Electron Microscopy (TEM, Tecnai F20) were used to probe the microstructure.

Raman spectroscopy (inVia Renishaw Raman microscope, λ = 633 nm) was recorded in the range of 10 to 3200 cm^−1^. 25 accumulations were acquired for each sample to diminish the effect of background noise.

X-ray photoelectron spectroscopy (XPS, Thermofisher ESCALAB 250i) was employed with hemispherical electron analyser. Monochromatic Al Kα (15 keV) with constant pass energy were used as X-ray source. Obtained C1s spectra, after Shirley background subtraction, were deconvoluted into Gaussian-Lorentzian peaks. Following bond assignments were used: C=C sp2 (284.4–284.6 eV), C–C sp3 (284.9–285.3 eV), C–O (286.0–286.4 eV), >C=O (287.3–287.7 eV) and –C(=O)O (288.7–289.1 eV)^26,27^.

## Results

### Surface chemistry of CNT films

**Figure 2:**
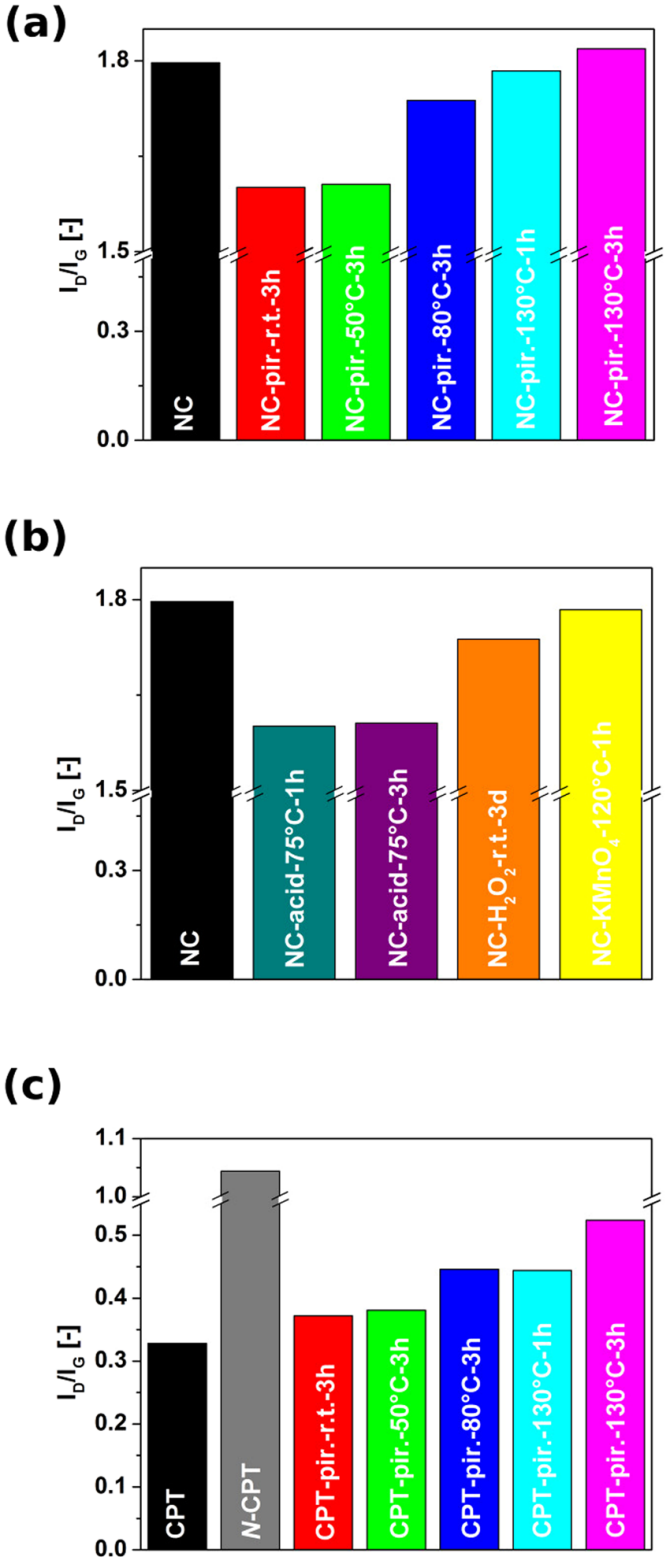
I_D_/I_G_ ratios of as-made and functionalized (**a**,**b**) NC and (**c**) CPT (as-made *N*-CPT is also shown) as measured by Raman spectroscopy.

Raman spectroscopy is a convenient tool to gauge surface chemistry of carbon nanomaterials. A ratio of intensities of sp^3^ defect-induced mode (D) to the mode of sp^2^ graphitic vibrations (G) reveals the degree of functionalization. We have subjected CNTs – technical grade NC as well as in-house made CPT – to a range of oxidizing treatments (methodology in Supplementary Information file). The goal was to find the most effective approach to introduce functional groups on the surface, which would increase the compatibility of CNTs with polymer matrices (measured indirectly as decrease in water/glycerol/epoxy resin contact angle). It was found that neat industrial grade NC had relatively high I_D_/I_G_ = 1.79, what is indicative of abundance of functional groups on the surface and various types of defects (Fig. 2a). We exposed this material to treatments with piranha solution at various temperatures for a specified amount of time. It was found that initially the I_D_/I_G_ ratios decrease to 1.60 and 1.62 for 3 h treatments at room temperature and 50 °C, respectively. We can expect that first the most defective species undergo their final oxidation step and are etched away from the surface, which effectively results in cleaning of the material^28^. As we increase the temperature, the I_D_/I_G_ ratios start to rise, which means that the predominant effect under these conditions is introduction of new functional groups. Reaction time study showed that treatment of NC CNTs for 1 h (I_D_/I_G_ = 1.78) was not as effective as that for 3 h (I_D_/I_G_ = 1.82), because with prolonged time further functional groups are added. Furthermore, reactions with H_2_SO_4_/HNO_3_ at 75 °C for 1 h (I_D_/I_G_ = 1.60) and 3 h (I_D_/I_G_ = 1.61) removed the most defective carbonaceous C-O moieties, but were not vigorous enough to functionalize the NC material beyond the starting degree (Fig. 2b). Next, prolonged stirring of CNTs in H_2_O_2_ solution even at room temperature can cause decrease in I_D_/I_G_^28,29^ by similar removal of oxygenated carbon functional groups. Here however the decrease in I_D_/I_G_ ratio is smaller (1.79 to 1.74) because 30 wt% H_2_O_2_ (aq) is much milder than H_2_SO_4_/HNO_3_ mixture.

Finally, the NC material was also treated with KMnO_4_ solution, which is known to cause significant oxidation^30^ and even nanotube unzipping under vigorous conditions^31^. I_D_/I_G_ level reached 1.78. Because piranha solution treatment at 130 °C for 3 h had the highest increase in I_D_/I_G_, therefore it was selected as the material for contact angle determination (denoted *O*-NC&EC). What regards CPT material, piranha solution gave a stepwise increase in the degree of functionalization with temperature and time of the treatment (Fig. 2c) eventually reaching I_D_/I_G_ = 0.524 (as compared with 0.328 for the starting material). The as-made CPT had much higher degree of structural perfection, so decrease in I_D_/I_G_ due to removal of highly defected species could not be observed as in the case of NC. Again, piranha solution treatment at 130 °C for 3 h had the highest increase in I_D_/I_G_, so such CPT material was chosen for further studies (denoted *O*-NC&EC). In addition, we have prepared N-doped CPT by adding nitrogen precursor to the reaction mixture (denoted *N*-CPT&EC). As can be seen that had a significant effect on the composition of the material and I_D_/I_G_, almost tripled eventually reaching 1.04 for as made material. Nitrogen presence distorts graphitic lattice and often introduces significant alterations to the structure of individual CNTs what results in significant elevation of the D mode^32,33^. Abundance of more hydrophilic nitrogen centers was the reason for selection of this material as well for further experiments.

### Removal of binder by thermal annealing

**Figure 3:**
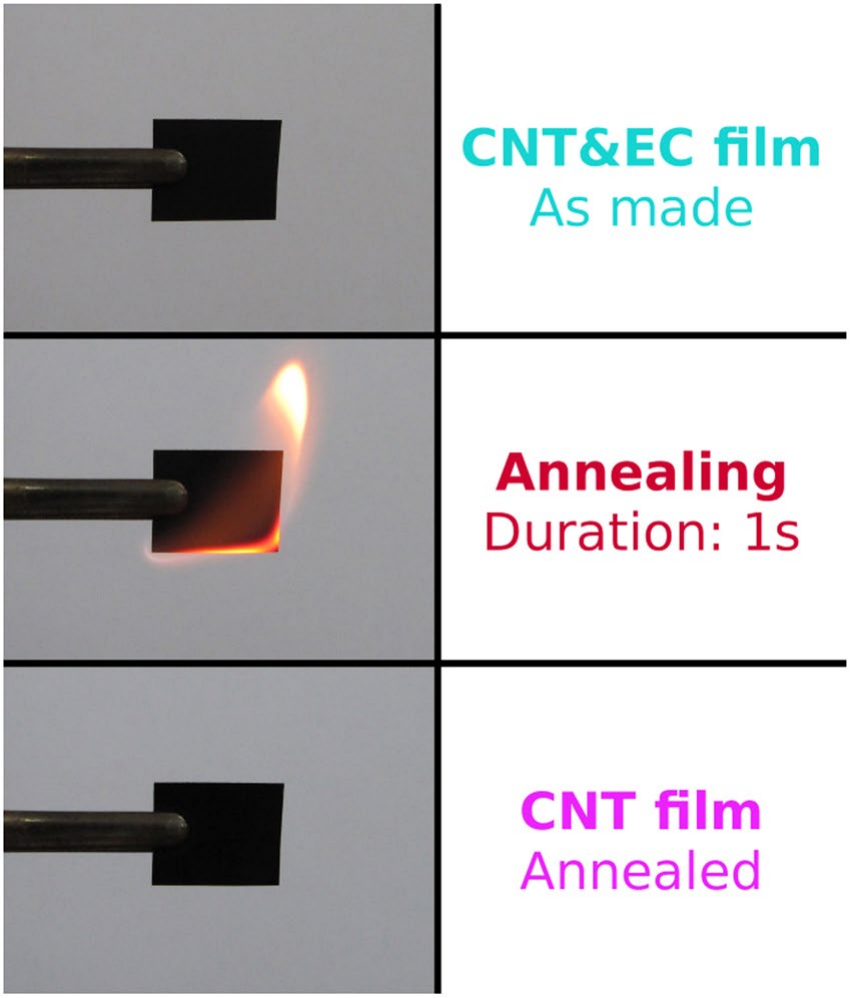
The process of flash annealing to remove EC binder from CNTs.

Figure 3 shows the process of removal of binder from CNT film. While 10 mm × 12 mm (additional 2 mm was added for the attachment of the film) specimen was securely kept with tweezers, it was ignited with a lighter to initiate the decomposition of EC. The whole process of annealing took only 1 second, during which a flame propagated through the film and combusted EC. Once the process was completed, the CNT film appeared very similar and, most importantly, it preserved its mechanical integrity^25^. The process of flash annealing is presented in the Supplementary Video 1.

It is important to stress that EC is ideal for making temporary scaffolding for CNT formation because its thermal removal does not leave residue as proven by thermogravimetric analysis in a flow of air^25^.

**Figure 4:**
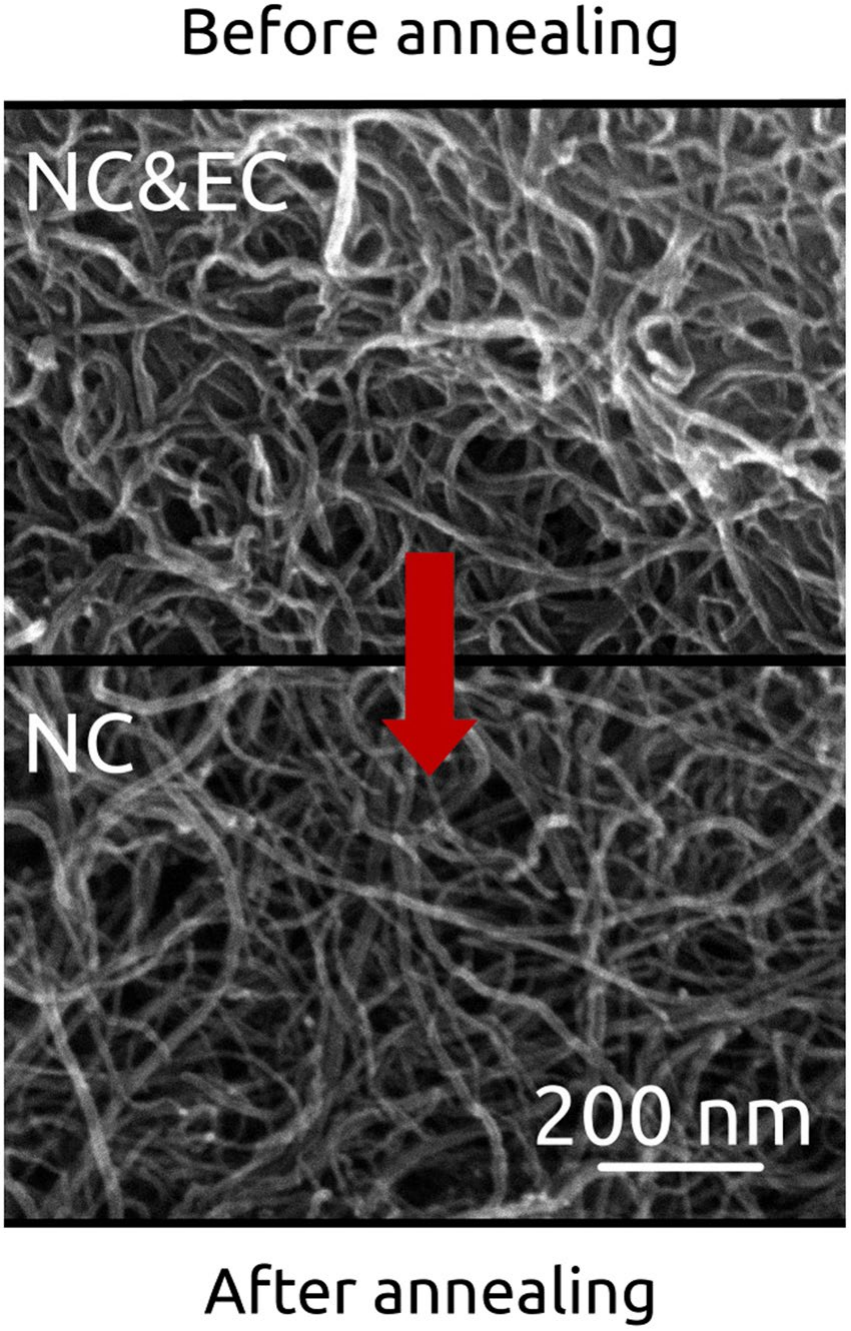
Microstructure of NC films before and after annealing as observed by SEM.

We have also carried out examination by SEM to demonstrate the influence of annealing on the microstructure of the material (Fig. 4). Flash removal of EC does not cause deterioration to the film microstructure. The voids between individual CNTs/their bundles were found to have relatively unaffected size. Moreover, no signs of obvious carbonaceous, non-CNT deposits, could be observed before and after annealing.

Although the effect shown is based on NC material, the conclusions are valid for films built from other types of CNTs^25^ as well.

**Figure 5:**
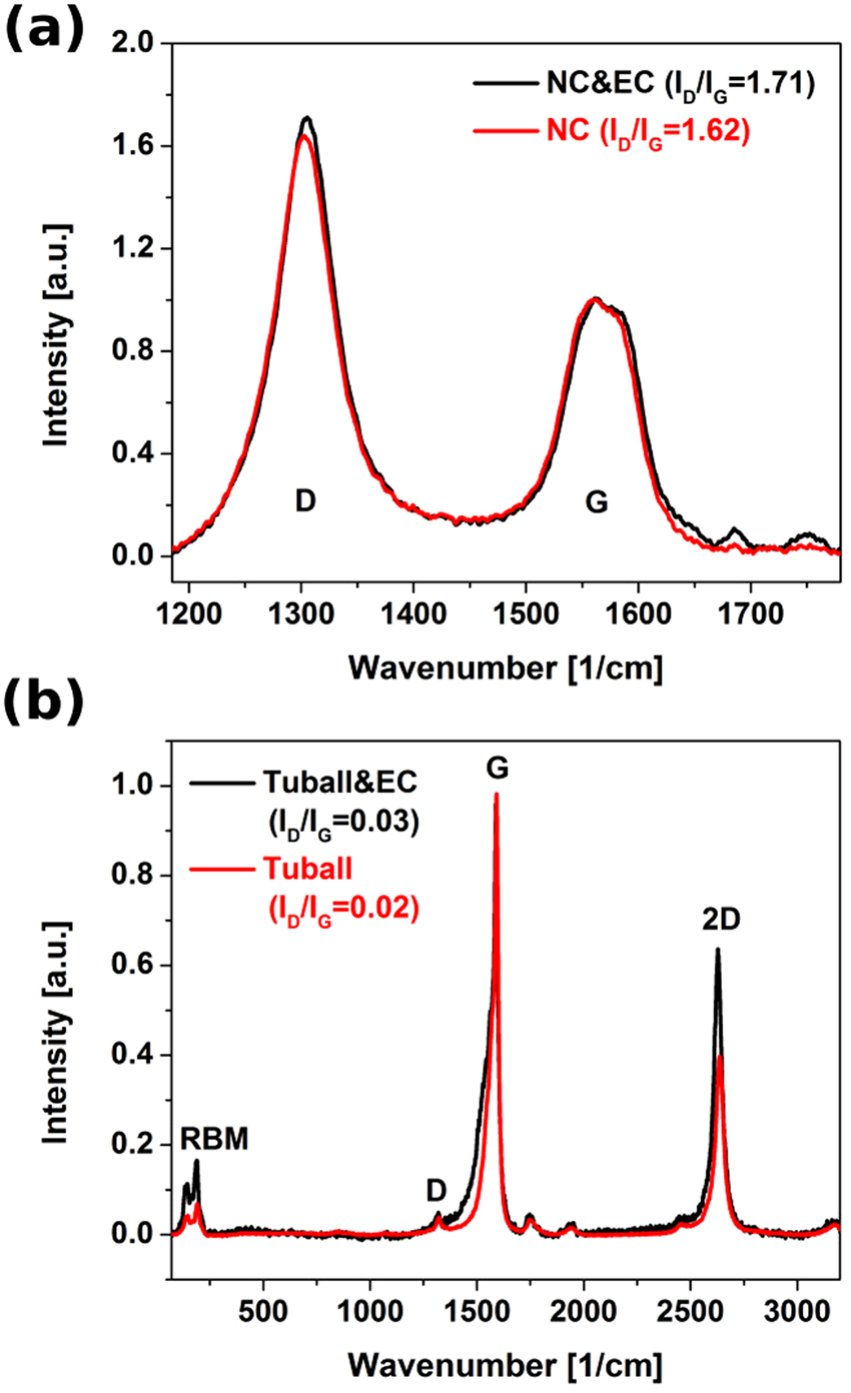
I_D_/I_G_ ratios of CNT films before and after thermal removal of EC assembled from (**a**) NC (technical-grade MWCNTs) and (**b**) Tuball (SWCNTs of highly pure structure) as measured by Raman spectroscopy.

From the composition point of view, thermal annealing was also found not to introduce any new functional groups on the surface. I_D_/I_G_ ratio of NC films actually decreased slightly from 1.71 to 1.62 because the most defective carbon-oxygen based species were removed from the surface as a consequence of the rapid exposure to high temperature (Fig. 5a). It is important now to give a proof for further contact angle considerations that even for highly crystalline CNTs the flash annealing does not oxidize the surface of the material if the process is carried out for a short amount of time. SWCNTs are much less thermally stable than DWCNTs or MWCNTs^34^, therefore they are the perfect “litmus paper” to test this hypothesis.

**Figure 6:**
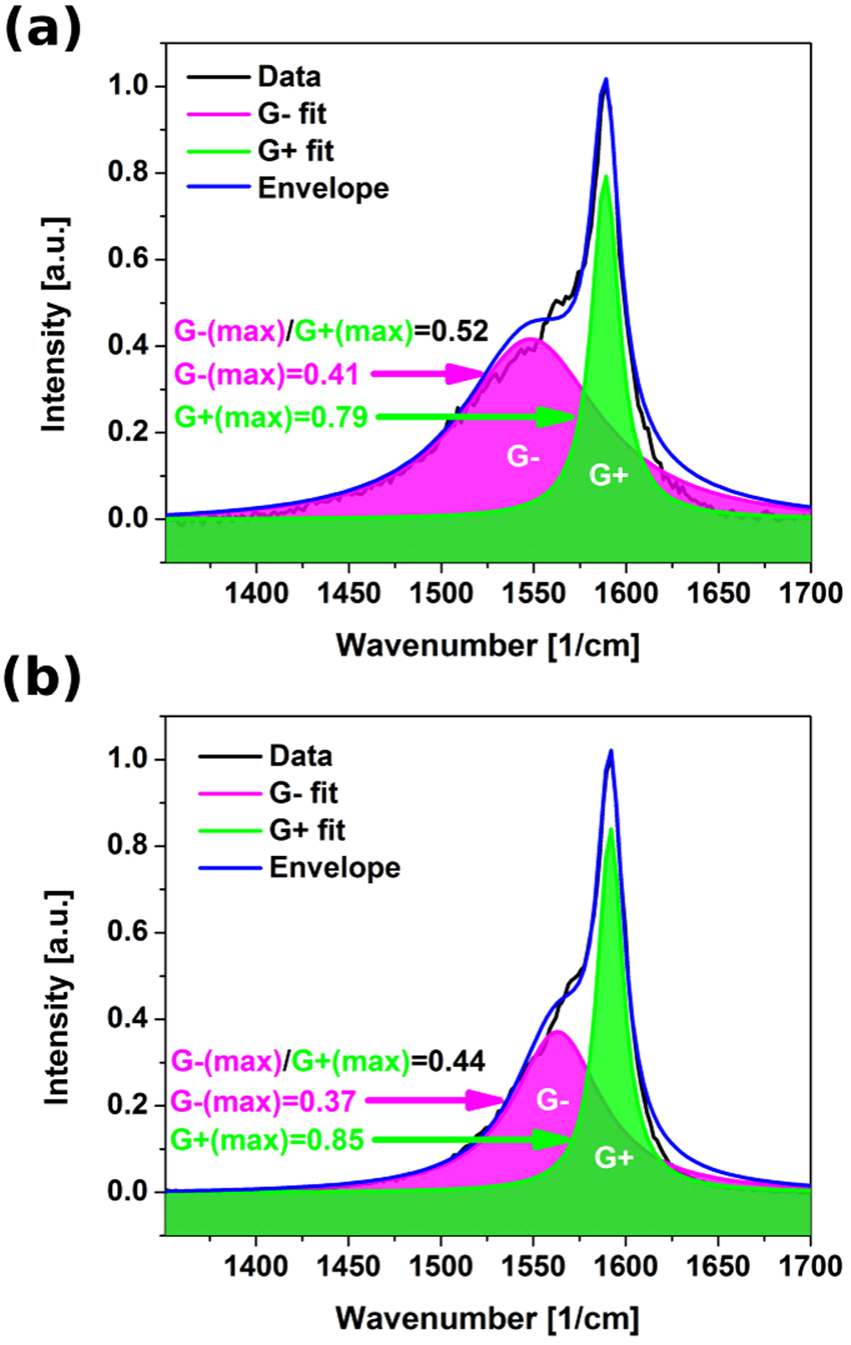
The influence of thermal annealing on the intensity of G− and G+ mode of SWCNTs. (**a**) Before annealing, (**b**) after annealing as measured by Raman spectroscopy.

Even SWCNTs of very high degree of pristinity (I_D_/I_G_ = 0.03) did not deteriorate during the course of 1s-long thermal annealing, but in fact we observed mild improvement to their crystallinity (Fig. 5b). I_D_/I_G_ was reduced down to 0.02. The G+ mode narrowed after the treatment (I_G−_/I_G+_ changed from 0.52 to 0.44), what indicates that the composition of the material became more homogeneous (Fig. 6)^35^.

**Figure 7:**
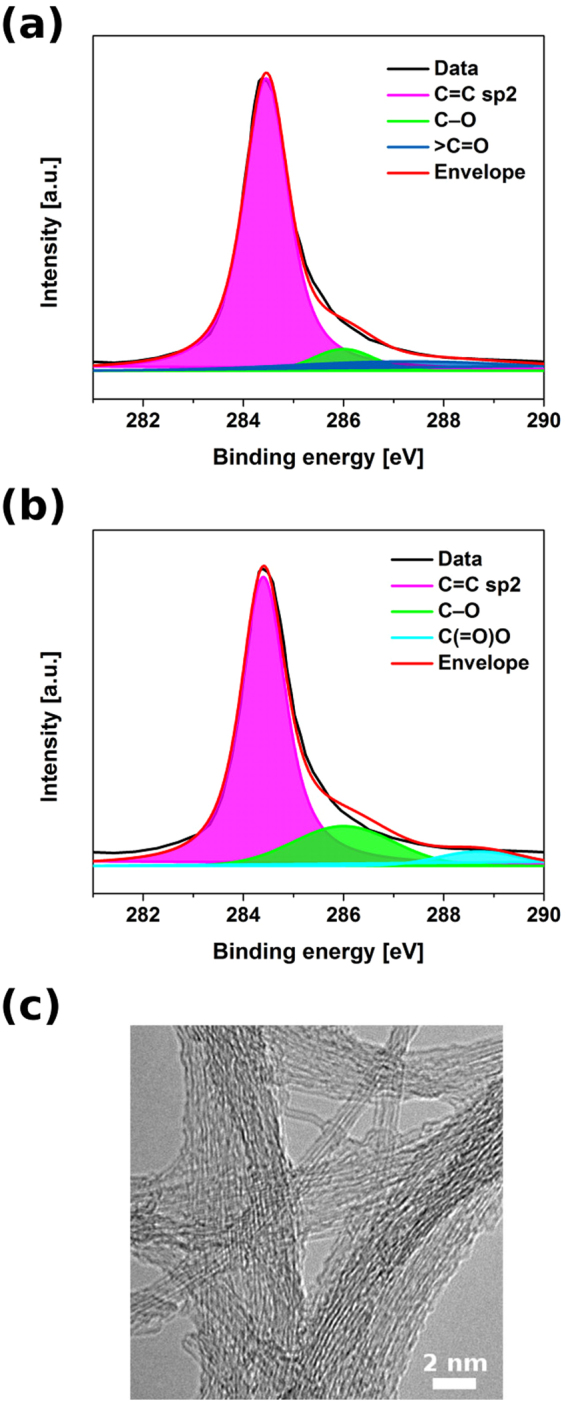
XPS spectra (C1s peaks) of (**a**) Tuball powder and (**b**) flash annealed CNT film from Tuball powder. (**c**) TEM micrograph of flash annealed CNT film from Tuball powder.

Closer investigation of the surface chemistry by XPS revealed additional information about the process of flash annealing (Fig. 7a,b). We observed a slight increase of the content of oxygen-containing functional groups. The recorded spectra also give insight into the underlying mechanism responsible for improved wettability. Hydroxyl and carbonyl groups are present in the parent material. Short exposure to high temperature seems to elevate the level of hydroxyl groups, convert some of them to carboxyl groups and eliminate the carbonyl groups by oxidizing them to CO_2_ effectively stripping these functional groups off from a CNT. Such rearrangements change the Hansen solubility parameters of CNTs, which makes them more hydrophilic^36,37^.

What regards the microstructure after annealing (Fig. 7c), no signs of deterioration have been detected. The film is predominantly made of single-wall CNT bundles with uniform diameter distribution. The lack of carbonaceous contaminants is reflected in notably low I_D_/I_G_ ratios.

### Contact angle measurements

**Figure 8:**
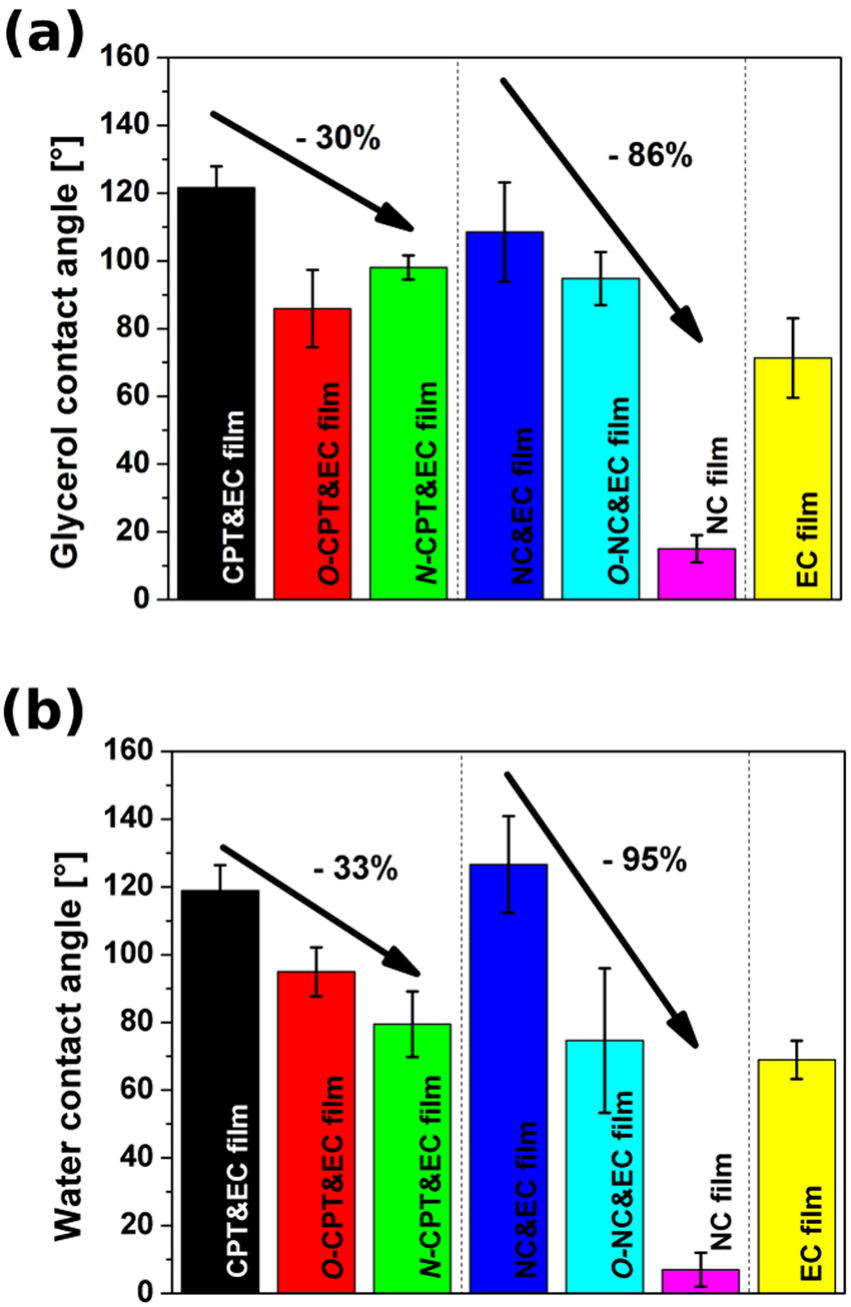
(**a**) Glycerol and (**b**) water initial contact angles of as-made and processed NC and CPT CNT films with and without EC. Contact angle of EC film is also given as reference.

We wanted to evaluate how the composition and surface chemistry of CNT films influences their wettability. Figure 8 shows the results of measurement of contact angle (glycerol, water) for various treated and untreated CNT films. We started with glycerol that has got three orders of magnitude higher viscosity than water^38^, but slightly lower surface tension 64 mJ/m^2^ (as compared with 72.8 mJ/m^2^ for water)^39^. As-made CPT&EC and NC&EC films had on average 122° and 109° glycerol contact angles, respectively (Fig. 8a). There is limited information about glycerol contact angle on CNT macroassemblies in the literature, but the consensus is that it is smaller than that of water^39,40^ or that it even wets CNTs completely^41^. In our case the glycerol contact angles for unfunctionalized films are relatively high. Introduction of functional groups by oxidation of CPT and NC reduced contact angles down to 86° (*O*-CPT&EC film) and 95° (*O*-NC&EC film), respectively. Moreover, *in situ* nitrogen doped CPT (*N*-CPT&EC film) had contact angle reduced to 98°. The presence of nitrogen in the sp^2^ carbon lattice increases the hydrophilic character of CNTs^42^ because *N*-doped CNTs are more polar than neat CNTs. In a simplified way, we can consider *N*-doped CNTs as “*quasi*-copolymer” of benzene and pyridine, which together form a polycyclic superstructure seamlessly wound into a tubule. Taking into the account the relative static permittivity as a measure of substance polarity, the more chemically doped pyridine-like nitrogen atoms in a CNT the more polar it becomes (ε(benzene) = 2.27, ε(pyridine) = 12.40^43^). The higher the polarity the more favorable the interaction of a substance with hydrophilic solvents such as glycerol or water.

It has to be noted that all of the described samples both for CPT and NC contained EC, which influenced the value of the glycerol contact angle. As-made unprocessed CNTs can have water contact angles as high as 154°^44^, which was counteracted here by the presence of EC to a certain extent. As a reference, we prepared a film from EC and the glycerol contact angle was measured to be 71°, so EC effectively decreased the contact angle as expected. We wanted to find out how removal of EC by flash annealing would affect the wettability of CNT films. As it turned out once the polymer binder is removed, the CNT films became superhydrophilic. Contact angle decreased by 86% from 122° to 15°. Even viscous solvent such as glycerol was rapidly absorbed by the NC-based CNT film.

Next, we selected water for testing of CNT films wettability (Fig. 8b). Water contact angles of untreated CPT&EC and NC&EC films had on average 119° and 127°, respectively. The values are relatively close for those measured with glycerol with the exception that the water contact angle for NC film is higher than 109° as recorded using glycerol droplets. Water contact angle of EC film 69° matched the literature data^45^. What regards surface functionalization by oxidation, water contact angle also decreased. In the case of *O*-CPT&EC film the change was less notable (95°) than for *O*-NC&EC film (75°). Furthermore, *in situ* doping of CNT carpet with nitrogen (*N*-CPT&EC film) once again improved the wettability by 33% (80°). However, the most striking results come again from NC film sample that was freed from EC. We estimate water contact angle of samples from this treatment to be about 7°. Relatively big standard deviation stem from the difficulty in precise estimation of water contact angle at this level. On average we observed 95% reduction from the initial NC&EC film water contact angle.

**Figure 9:**
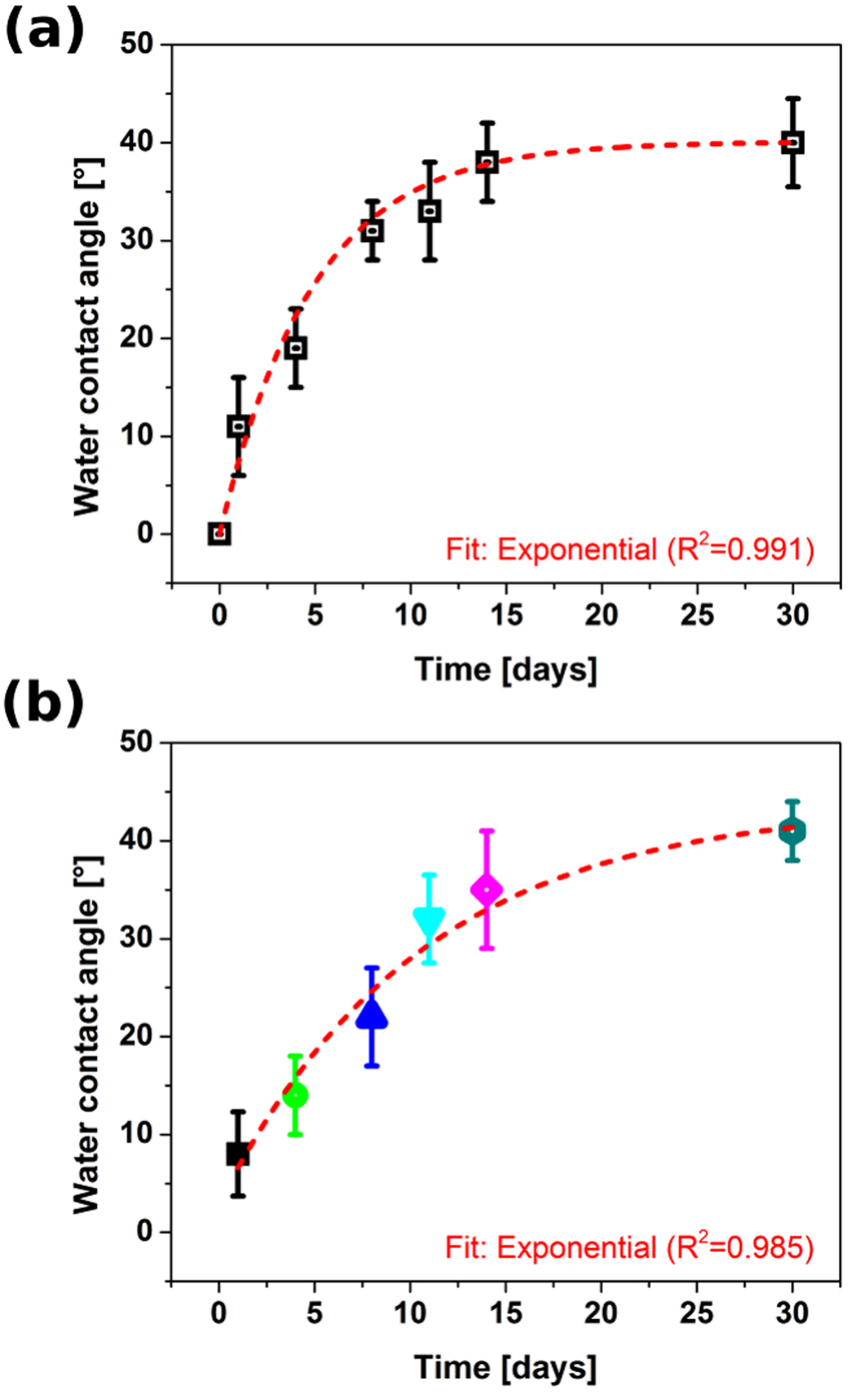
Evolution of water contact angle for annealed NC CNT films: (**a**) repeated on the same subset of samples and (**b**) carried out using fresh samples for each measurement point.

To verify how permanent this effect is, we carried out water contact angle measurements as a function of time. First, the same set of NC film samples was subjected to repeated water contact angle measurements (Fig. 9a). At t = 5 min, the first measurement point, the CNT films absorbed all the water therefore we assumed the contact angle of 0°. With repeated application of water droplet at given time intervals, the contact angle slowly restored at an exponential pace. After one month, it eventually reached 40°, which appears to be the limit.

There are reports that upon heating or prolonged exposure to ambient humidity the water contact angle of transiently hydrophilic CNT films may reverse^46,47^. UV/ozone^47^, plasma^48^ or heating^46,49–53^ is able to change the character of the surface between hydrophobic and hydrophilic, but once the effect fades, the water contact angle often restores to its original value. It is interesting that a slight increase in content and redistribution of types of oxygen functionalities (as indicated by XPS) made the NC film relatively hydrophilic (40°). In our case however, the water contact angle did not come back to its initial value. The recovery time of was also slower at this humidity by two orders of magnitude as compared with published results^46^. The partial reversal of the effect could be justified by desorption of weakly bonded C–O components, which can improve water affinity of the CNT material^54^.

Furthermore, we wanted to find out whether annealed NC films stored under the ambient humidity conditions would be more hydrophilic than repeatedly treated annealed NC films with water droplets (Fig. 9b). It appears that initially the fresh films are indeed more hydrophilic, but only slightly. As the time progresses, fresh NC films also tend to about 40° water contact angle.

To confirm that the method of making the CNT films hydrophilic is applicable to real life case applications, we carried out Bisphenol-A contact angle measurements on them. Epoxy resins (with appropriate hardener) are widely used components to make high-performance polymer matrix composites based on nanocarbon materials^55–59^. Although CNT reinforcement can significantly improve the mechanical properties^56,57,60^, the material is often hard to integrate with polymer due to its inherently hydrophobic nature^60–63^. We would like to show that commonly employed oxidation, which introduces necessary functional groups often at the performance expense, is not necessary. As it has been recently reported, a few pairs of topological defects can have a dramatic influence on the tensile strength, therefore functionalization level must be kept to the absolute minimum^17^.

**Figure 10:**
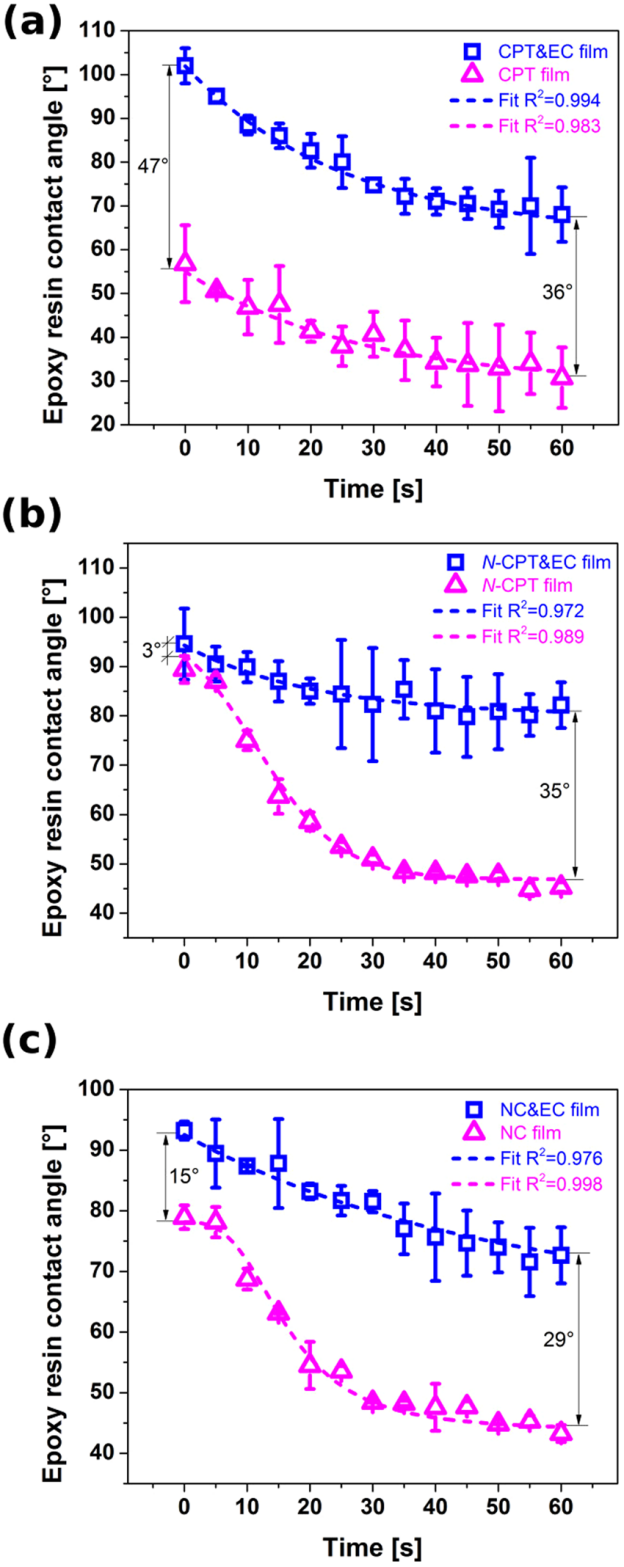
Evolution of epoxy resin contact angle for CNT films with and without EC based on (**a**) CPT, (**b**) *N*-CPT and (**c**) NC CNTs.

There is an evident difference between the evolution of epoxy resin contact angle between CNT films with and without EC binder (Fig. 10). All of the as made CNT&EC films have an initial contact angle of about 100°, which slowly transforms between Cassie state and the Wenzel state as the droplet spreads out and wets the surface^64^. After 60 minutes, the contact angle reaches 68°, 82° and 73° for CPT&EC, *N*-CPT&EC and NC&EC films, respectively. The wetting with epoxy resin was much better when the films were previously subjected to flash annealing to remove the EC. In such case, the contact angle for CPT, *N*-CPT and NC films was as low as 31°, 45° and 43°, respectively. As compared with the final contact angle values for the un-annealed samples we reach up to 36° apparent improvement without making any negative impact on CNT properties.

**Figure 11:**
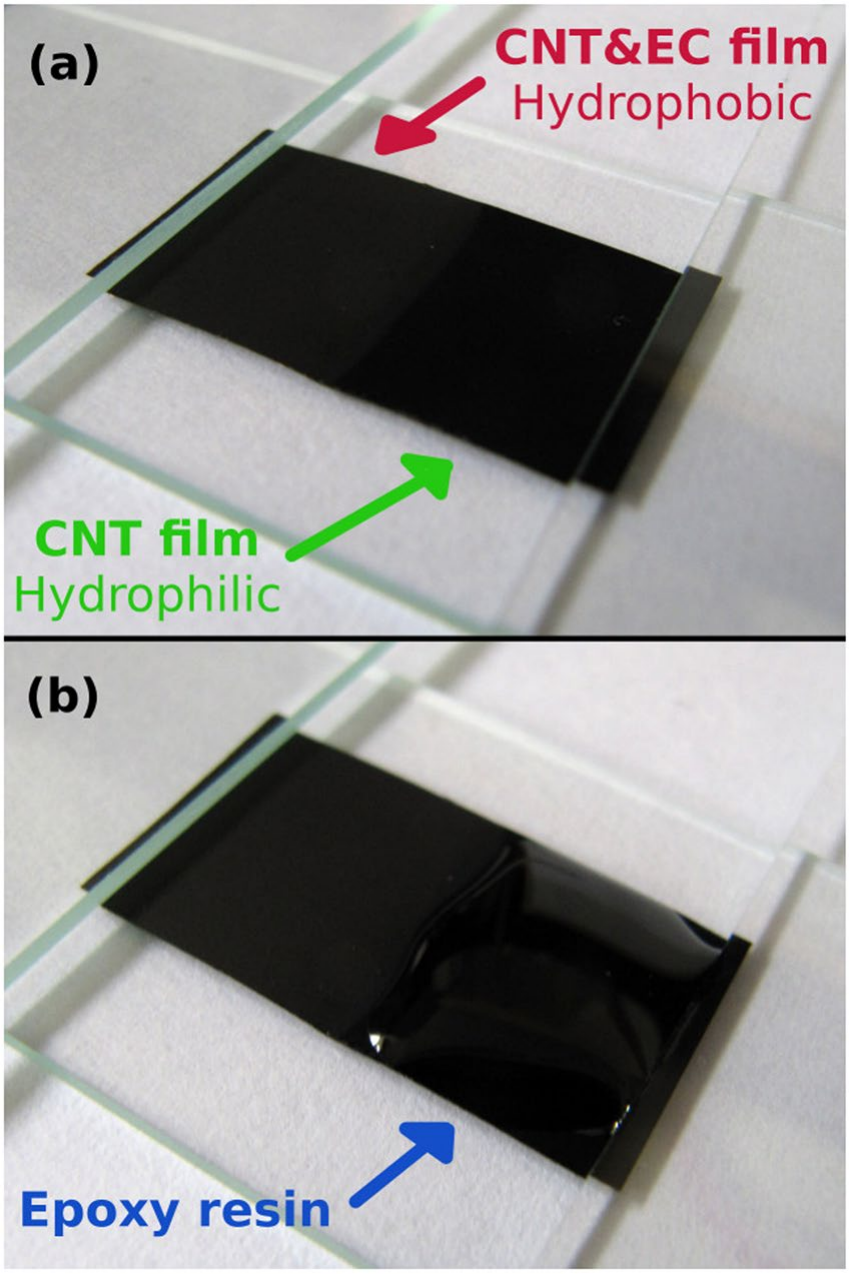
(**a**) CNT film composed of hydrophobic (with EC) and hydrophilic half (without EC - annealed), (**b**) final state of epoxy resin droplet deposited onto the hydrophilic half.

To make a final illustration how the surface character of CNT film changes from hydrophobic to hydrophilic after annealing, we prepared a film, half of which we annealed. It was patterned by covering one half of the CNT&EC film with a microscope glass slide during annealing, which protected EC from combustion (Fig. 11a). EC is a white solid, so the half with EC appears greyish as compared with the one without EC, which is black as it is composed of just CNTs. We deposited a droplet of epoxy resin onto the hydrophilic part and observed how its shape developed as time progressed. It was striking to see that as the droplet spread out on the material, it did stop at the hydrophobic-hydrophilic border between the two halves of the CNT film (Fig. 11b). Only the hydrophilic part was properly wetted. The proposed method has been successfully validated again.

## Conclusions

We presented a method how to prepare hydrophilic CNT films without making deterioration to the microstructure or composition of individual CNTs. Common approached focused on oxidation with harsh chemicals used for this purpose is not only a burden to the environment, requires utmost level of safety, but it heavily affects the properties of the CNTs. From the structural point of view, under such conditions, the CNTs are also often heavily fragmented and opened at the ends. Our technique of making CNT films with subsequent flash annealing to remove the binder is fully scalable as CNT films of any size can be made. Moreover, as revealed by the experiments, it gives much better results in terms of contact angle as compared with oxidizing treatments based on concentrated acid mixtures, piranha solution, *etc*. The method is very universal and works with any type of CNTs ranging from highly defected technical grade multi-wall CNT materials to very pristine single-wall CNTs.

Due to a much higher affinity towards hydrophilic media, the CNT films are more compatible with traditional interface materials such as polymers. For instance, in this study, CNT films presented excellent interaction with D.E.R. 332 epoxy resin, which is commonly used for making structural and/or electrical laminates. Because the process of making these hydrophilic CNT films is highly scalable, we can imagine integration with polymer matrices and application of such composites in a wide range of applications. Most immediate areas of implementation involve composites for mechanical reinforcement, EMI shielding or lightning protection. Proof of concept of such specialized applications would pave the way for CNT film integration in consumer products for the gain in everyday life.

Lastly, the novel ability to pattern the CNT macroassembly with hydrophobic and hydrophilic parts where required, enables formation of smarter multifunctional nanomaterials.

## Electronic supplementary material

Supplementary Video

Supplementary Information file

## References

[1] Pop E, Mann D, Wang Q, Goodson K, Dai H (2006). Thermal Conductance of an Individual Single-Wall Carbon Nanotube above Room Temperature. Nano Letters.

[2] Thostenson ET, Li C, Chou T-W (2005). Nanocomposites in context. Composites Science and Technology.

[3] Balandin AA (2008). Superior Thermal Conductivity of Single-Layer Graphene. Nano Letters.

[4] Koziol KK, Janas D, Brown E, Hao L (2017). Thermal properties of continuously spun carbon nanotube fibres. Physica E: Low-dimensional Systems and Nanostructures.

[5] Hong S, Myung S (2007). Nanotube Electronics: A flexible approach to mobility. Nat Nano.

[6] Stankovich, S. *et al*. Graphene-based composite materials. *Nature* **442**, 282–286, http://www.nature.com/nature/journal/v442/n7100/suppinfo/nature04969_S1.html (2006). 10.1038/nature04969 16855586

[7] Lekawa-Raus A, Gizewski T, Patmore J, Kurzepa L, Koziol KK (2017). Electrical transport in carbon nanotube fibres. Scripta Materialia.

[8] Yu M-F (2000). Strength and Breaking Mechanism of Multiwalled Carbon Nanotubes Under Tensile Load. Science.

[9] Lee C, Wei X, Kysar JW, Hone J (2008). Measurement of the Elastic Properties and Intrinsic Strength of Monolayer Graphene. Science.

[10] Janas D, Czechowski N, Krajnik B, Mackowski S, Koziol KK (2013). Electroluminescence from carbon nanotube films resistively heated in air. Applied Physics Letters.

[11] Liu J, Wright AR, Zhang C, Ma Z (2008). Strong terahertz conductance of graphene nanoribbons under a magnetic field. Applied Physics Letters.

[12] Janas D, Kreft SK, Koziol KKK (2015). Steam reforming on reactive carbon nanotube membranes. Journal of Industrial and Engineering Chemistry.

[13] Janas D, Koziol KK (2013). Rapid electrothermal response of high-temperature carbon nanotube film heaters. Carbon.

[14] An L, Friedrich CR (2012). Measurement of contact resistance of multiwall carbon nanotubes by electrical contact using a focused ion beam. Nuclear Instruments and Methods in Physics Research Section B: Beam Interactions with Materials and Atoms.

[15] Nicolo’ C (2011). Measuring the electrical resistivity and contact resistance of vertical carbon nanotube bundles for application as interconnects. Nanotechnology.

[16] Marconnet AM, Panzer MA, Goodson KE (2013). Thermal conduction phenomena in carbon nanotubes and related nanostructured materials. Reviews of Modern Physics.

[17] Zhu L, Wang J, Ding F (2016). The Great Reduction of a Carbon Nanotube’s Mechanical Performance by a Few Topological Defects. ACS Nano.

[18] Cho S-G, Ko K-C (2010). Surface free energy and super-hydrophobic coating of multi-walled carbon nanotubes by 3:1 TMCS/toluene glow discharge plasma under low pressure. Thin Solid Films.

[19] Martinez-Rubi Y (2012). Tailored SWCNT functionalization optimized for compatibility with epoxy matrices. Nanotechnology.

[20] Mahmood, N., Islam, M., Hameed, A. & Saeed, S. Polyamide 6/Multiwalled Carbon Nanotubes Nanocomposites with Modified Morphology and Thermal Properties. *Polymers* **5**, doi:10.3390/polym5041380 (2013).

[21] Liu Y-L (2016). Effective approaches for the preparation of organo-modified multi-walled carbon nanotubes and the corresponding MWCNT/polymer nanocomposites. Polym J.

[22] Hsiao A-E, Tsai S-Y, Hsu M-W, Chang S-J (2012). Decoration of multi-walled carbon nanotubes by polymer wrapping and its application in MWCNT/polyethylene composites. Nanoscale Research Letters.

[23] Boncel S, Pattinson SW, Geiser V, Shaffer MSP, Koziol KKK (2014). En route to controlled catalytic CVD synthesis of densely packed and vertically aligned nitrogen-doped carbon nanotube arrays. Beilstein Journal of Nanotechnology.

[24] Singh C, Shaffer M, Kinloch I, Windle A (2002). Production of aligned carbon nanotubes by the CVD injection method. Physica B: Condensed Matter.

[25] Janas D, Rdest M, Koziol KKK (2017). Free-standing films from chirality-controlled carbon nanotubes. Materials & Design.

[26] Ago H (1999). Work Functions and Surface Functional Groups of Multiwall Carbon Nanotubes. The Journal of Physical Chemistry B.

[27] Yang-Chih H, Chih-Chieh W, Chueh L, Chi-Chung K, Tsong-Pyng P (2012). Deposition of platinum on oxygen plasma treated carbon nanotubes by atomic layer deposition. Nanotechnology.

[28] Janas D, Herman AP, Boncel S, Koziol KKK (2014). Swift modification of resistively heated carbon nanotube films by the action of hydrogen peroxide. Materials Letters.

[29] Peng Y, Liu H (2006). Effects of Oxidation by Hydrogen Peroxide on the Structures of Multiwalled Carbon Nanotubes. Industrial & Engineering Chemistry Research.

[30] Wepasnick KA (2011). Surface and structural characterization of multi-walled carbon nanotubes following different oxidative treatments. Carbon.

[31] Kosynkin, D. V. *et al*. Longitudinal unzipping of carbon nanotubes to form graphene nanoribbons. *Nature* **458**, 872–876, http://www.nature.com/nature/journal/v458/n7240/suppinfo/nature07872_S1.html (2009). 10.1038/nature07872 19370030

[32] Daems N, Sheng X, Vankelecom IFJ, Pescarmona PP (2014). Metal-free doped carbon materials as electrocatalysts for the oxygen reduction reaction. Journal of Materials Chemistry A.

[33] Arjmand M, Sundararaj U (2015). Effects of Nitrogen Doping on X-band Dielectric Properties of Carbon Nanotube/Polymer Nanocomposites. ACS Applied Materials & Interfaces.

[34] Janas D, Cabrero-Vilatela A, Bulmer J, Kurzepa L, Koziol KK (2013). Carbon nanotube wires for high-temperature performance. Carbon.

[35] Telg H (2008). G− and G+ in the Raman spectrum of isolated nanotube: a study on resonance conditions and lineshape. physica status solidi (b).

[36] Ma J, Larsen RM (2013). Effect of Surface Modification on the Hansen Solubility Parameters of Single-Walled Carbon Nanotubes. Industrial & Engineering Chemistry Research.

[37] Bergin SD (2009). Multicomponent Solubility Parameters for Single-Walled Carbon Nanotube−Solvent Mixtures. ACS Nano.

[38] Cheng N-S (2008). Formula for the Viscosity of a Glycerol−Water Mixture. Industrial & Engineering Chemistry Research.

[39] Barber AH, Cohen SR, Wagner HD (2005). External and internal wetting of carbon nanotubes with organic liquids. Physical Review B.

[40] Mattia D, Bau HH, Gogotsi Y (2006). Wetting of CVD Carbon Films by Polar and Nonpolar Liquids and Implications for Carbon Nanopipes. Langmuir.

[41] De Nicola, F. *et al*. Multi-Fractal Hierarchy of Single-Walled Carbon Nanotube Hydrophobic Coatings. **5**, 8583, doi:10.1038/srep08583https://www.nature.com/articles/srep08583#supplementary-information (2015). 10.1038/srep08583 PMC4341200 25716718

[42] Phao N, Nxumalo EN, Mamba BB, Mhlanga SD (2013). A nitrogen-doped carbon nanotube enhanced polyethersulfone membrane system for water treatment. Physics and Chemistry of the Earth, Parts A/B/C.

[43] https://depts.washington.edu/eooptic/linkfiles/dielectric_chart%5B1%5D.pdf.

[44] Francesco De,N (2015). Super-hydrophobic multi-walled carbon nanotube coatings for stainless steel. Nanotechnology.

[45] Bartell FE, Ray BR (1952). Wetting Characteristics of Cellulose Derivatives. I. Contact Angles Formed by Water and by Organic Liquids1. Journal of the American Chemical Society.

[46] Yang J, Zhang Z, Men X, Xu X, Zhu X (2011). Thermo-responsive surface wettability on a pristine carbon nanotube film. Carbon.

[47] Wang HZ (2010). Reversible transformation of hydrophobicity and hydrophilicity of aligned carbon nanotube arrays and buckypapers by dry processes. Carbon.

[48] Singh N (2016). Chitin and carbon nanotube composites as biocompatible scaffolds for neuron growth. Nanoscale.

[49] Muthiah P, Hoppe SM, Boyle TJ, Sigmund W (2011). Thermally Tunable Surface Wettability of Electrospun Fiber Mats: Polystyrene/Poly(N-isopropylacrylamide) Blended versus Crosslinked Poly[(N-isopropylacrylamide)-co-(methacrylic acid)]. Macromolecular Rapid Communications.

[50] Wang J, Liu M, Ma R, Wang Q, Jiang L (2014). In Situ Wetting State Transition on Micro- and Nanostructured Surfaces at High Temperature. ACS Applied Materials & Interfaces.

[51] Drelich J, Chibowski E, Meng DD, Terpilowski K (2011). Hydrophilic and superhydrophilic surfaces and materials. Soft Matter.

[52] Guo F, Guo Z (2016). Inspired smart materials with external stimuli responsive wettability: a review. RSC Advances.

[53] Janas D, Sundaram R, Koziol KKK (2012). Surface modification of directly spun carbon nanotube films. Materials Letters.

[54] He L, Karumuri A, Mukhopadhyay SM (2017). Wettability tailoring of nanotube carpets: morphology-chemistry synergy for hydrophobic-hydrophilic cycling. RSC Advances.

[55] Sandler JKW, Kirk JE, Kinloch IA, Shaffer MSP, Windle AH (2003). Ultra-low electrical percolation threshold in carbon-nanotube-epoxy composites. Polymer.

[56] Allaoui A, Bai S, Cheng HM, Bai JB (2002). Mechanical and electrical properties of a MWNT/epoxy composite. Composites Science and Technology.

[57] Gojny FH, Wichmann MHG, Köpke U, Fiedler B, Schulte K (2004). Carbon nanotube-reinforced epoxy-composites: enhanced stiffness and fracture toughness at low nanotube content. Composites Science and Technology.

[58] Yue L, Pircheraghi G, Monemian SA, Manas-Zloczower I (2014). Epoxy composites with carbon nanotubes and graphene nanoplatelets – Dispersion and synergy effects. Carbon.

[59] Sandler J (1999). Development of a dispersion process for carbon nanotubes in an epoxy matrix and the resulting electrical properties. Polymer.

[60] Park SH, Bandaru PR (2010). Improved mechanical properties of carbon nanotube/polymer composites through the use of carboxyl-epoxide functional group linkages. Polymer.

[61] Park M (2011). Improved binding between copper and carbon nanotubes in a composite using oxygen-containing functional groups. Carbon.

[62] Špitalský Z, Krontiras CA, Georga SN, Galiotis C (2009). Effect of oxidation treatment of multiwalled carbon nanotubes on the mechanical and electrical properties of their epoxy composites. Composites Part A: Applied Science and Manufacturing.

[63] Yoonessi M, Lebrón-Colón M, Scheiman D, Meador MA (2014). Carbon Nanotube Epoxy Nanocomposites: The Effects of Interfacial Modifications on the Dynamic Mechanical Properties of the Nanocomposites. ACS Applied Materials & Interfaces.

[64] Wirth CT, Hofmann S, Robertson J (2008). Surface properties of vertically aligned carbon nanotube arrays. Diamond and Related Materials.

