# Supplementary material for: Unexpectedly strong hydrophilic character of free-standing thin films from carbon nanotubes
Source: Sci Rep. 2017 Sep 25;7:12274. doi: 10.1038/s41598-017-12443-y (PMC5612993; doi:10.1038/s41598-017-12443-y)
Supplement: Supplementary file 2 — Supplementary Information file [file 41598_2017_12443_MOESM2_ESM.pdf]

Electronic supplementary information

for

Unexpectedly strong hydrophilic character of free-standing thin films from carbon nanotubes

by

Dawid Janas[a,*], Grzegorz Stando[a]

[a] *Department of Chemistry, Silesian University of Technology, B. Krzywoustego 4, 44-100 Gliwice, Poland*

Corresponding author:

Tel.: + 48 32 2372958

E-mail address: Dawid.Janas@polsl.pl (Dawid Janas)

NC7000 treatments

Piranha solution

100 mg of CNT powder was added to 25 mL of freshly prepared piranha solution (30% H_2O_2 dripped into ice-cold 98% H_2SO_4, 7/3 V/V – the reverse procedure may produce explosive mixture). It was stirred on the hotplate for a specified time. The temperature was maintained by keeping the flask in a silicon oil bath. After the reaction was complete, the product was filtered and washed with distilled water under reduced pressure using Whatman 0.2 μm PTFE filters until the filtrate was at neutral pH. The following parameters were employed.

No.	Symbol	Temperature [°C]	Time [h]
1	NC-piranha-r.t.-3h	r.t.	3
2	NC-piranha-50°C-3h	50	3
3	NC-piranha-80°C-3h	80	3
4	NC-piranha-130°C-1h	130	1
5	NC-piranha-130°C-3h	130	3

Acid mixture

500 mg of CNT powder was added to 150 mL of concentrated H_2SO_4 (98%). Next, 50 mL of concentrated HNO_3 (68%) was slowly introduced. The mixture was put into a bath sonicator set at 75°C for a specified time. After the reaction was complete, the product was filtered and washed with distilled water under reduced pressure using Whatman 0.2 μm PTFE filters until the filtrate was at neutral pH. The following parameters were employed.

No.	Symbol	Temperature [°C]	Time [h]
1	NC-acid-r.t.-3h	75	1
2	NC-acid-50°C-3h	75	3

Hydrogen peroxide

250 mg of CNT powder was added to 30 mL of H_2O_2 solution (30%). The mixture was stirred for 72h. After 24h and 48h of reaction duration, 5 mL of fresh H_2O_2 solution was introduced. After the reaction was complete, the product was filtered and washed with distilled water and methanol under reduced pressure using Whatman 0.2 μm PTFE filters until the filtrate was at neutral pH. The following parameters were employed.

No.	Symbol	Temperature [°C]	Time [h]
1	NC-H_2O_2-r.t.-3d	r.t.	72

Potassium permanganate

250 mg of CNT powder was added to 42 mL of H_2SO_4 solution (0.5 M). Then, 4.125 g of $KMnO_4$ in 42 mL of 0.5 M H_2SO_4 solution was introduced dropwise. The mixture was refluxed for 3 hours. After the reaction was complete, the mixture was quenched with 10 mL of H_2O_2 solution. The product was filtered and washed with concentrated hydrochloric solution (to dissolve MnO_2) and distilled water under reduced pressure using Whatman 0.2 μm PTFE filters until the filtrate was at neutral pH. The following parameters were employed.

No.	Symbol	Temperature [°C]	Time [h]
1	NC-$KMnO_4$-r.t.-3h	120	3

CNT carpet treatments

Piranha solution

100 mg of CNT powder was added to 25 mL of freshly prepared piranha solution (33% H_2O_2 dripped into ice-cold 98% H_2SO_4, 7/3 V/V – the reverse procedure may produce explosive mixture). It was stirred on the hotplate for a specified time. The temperature was maintained by keeping the flask in a silicon oil bath. After the reaction was complete, the product was filtered and washed under with distilled water reduced pressure using Whatman 0.2 μm PTFE filters until the filtrate was at neutral pH. The following parameters were employed.

No.	Symbol	Temperature [°C]	Time [h]	
1	CPT-piranha -r.t.-3h	r.t.	3	
2	CPT-piranha-50°C-3h	50	3	
3	CPT-piranha-80°C-3h	80	3	
4	CPT-piranha-130°C-1h	130	1	
5	CPT-piranha-130°C-3h	130	3	⬅

Note: Arrows indicate selected treatment parameters that were used to produce CNT films for further analysis described in the main text.
